# Tautomerism as primary signaling mechanism in metal sensing: the case of amide group

**DOI:** 10.3762/bjoc.15.185

**Published:** 2019-08-08

**Authors:** Vera Deneva, Georgi Dobrikov, Aurelien Crochet, Daniela Nedeltcheva, Katharina M Fromm, Liudmil Antonov

**Affiliations:** 1 Institute of Organic Chemistry with Centre of Phytochemistry, Bulgarian Academy of Sciences, Sofia 1113, Bulgaria; 2 University of Fribourg, Department of Chemistry, Chemin du Musée 9, CH-1700 Fribourg, Switzerland

**Keywords:** amide group, azo dye, molecular sensor, sidearm, tautomerism

## Abstract

The concept for sensing systems using the tautomerism as elementary signaling process has been further developed by synthesizing a ligand containing 4-(phenyldiazenyl)naphthalene-1-ol as a tautomeric block and an amide group as metal capturing antenna. Although it has been expected that the intramolecular hydrogen bonding (between the tautomeric hydroxy group and the nitrogen atom from the amide group) could stabilize the pure enol form in some solvents, the keto tautomer is also observed. This is a result from the formation of intramolecular associates in some solvents. Strong bathochromic and hyperchromic effects in the visible spectra accompany the 1:1 formation of complexes with some alkaline earth metal ions.

## Introduction

The design of new organic sensing systems is an undividable part of the development of coordination chemistry [1]. Particularly chromophore ligands have been successfully utilized for colorimetric detection of the majority of metal ions as complex [2]. Some of them are used as standard tools in chelatometric titrations [3]. The design of specific ligands for alkali metal determination is still a challenge. In the case of alkaline earth metal ions, the reagents with reasonable selectivity are still not commonly accepted since they compete with transitional metal ions [4]. The discovery of crown ethers [5] and 3D-based ligands [6] unquestionably helped the development of natural ligand-supported metal investigations.

**Scheme 1:**
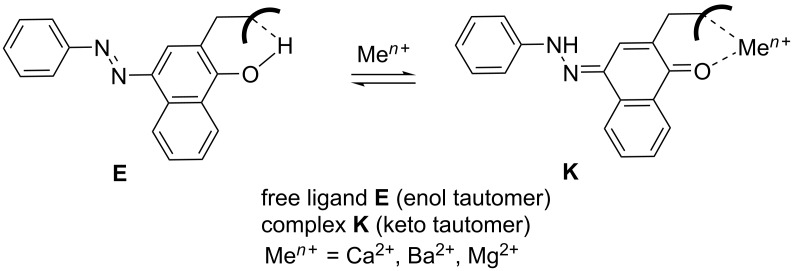
Conceptual idea for tautomeric metal sensing.

The ion recognition is based on the existence of two molecular states (ligand and complex) with different optical properties and a structure that allows fast transfer from the ligand to the complex upon addition of the desired metal ion [7]. The tautomeric proton exchange has the same properties when the equilibrium is switched from one to the other tautomer. The tautomerism can be controlled by metal ion addition, when an ionophore unit is implemented in the tautomeric backbone. The conceptual idea to achieve the pure enol tautomer through intramolecular hydrogen bonding with the ionophore [7–8] is shown in Scheme 1. The complex formation ejects the tautomeric proton and stabilizes the keto tautomer.

**Scheme 2:**
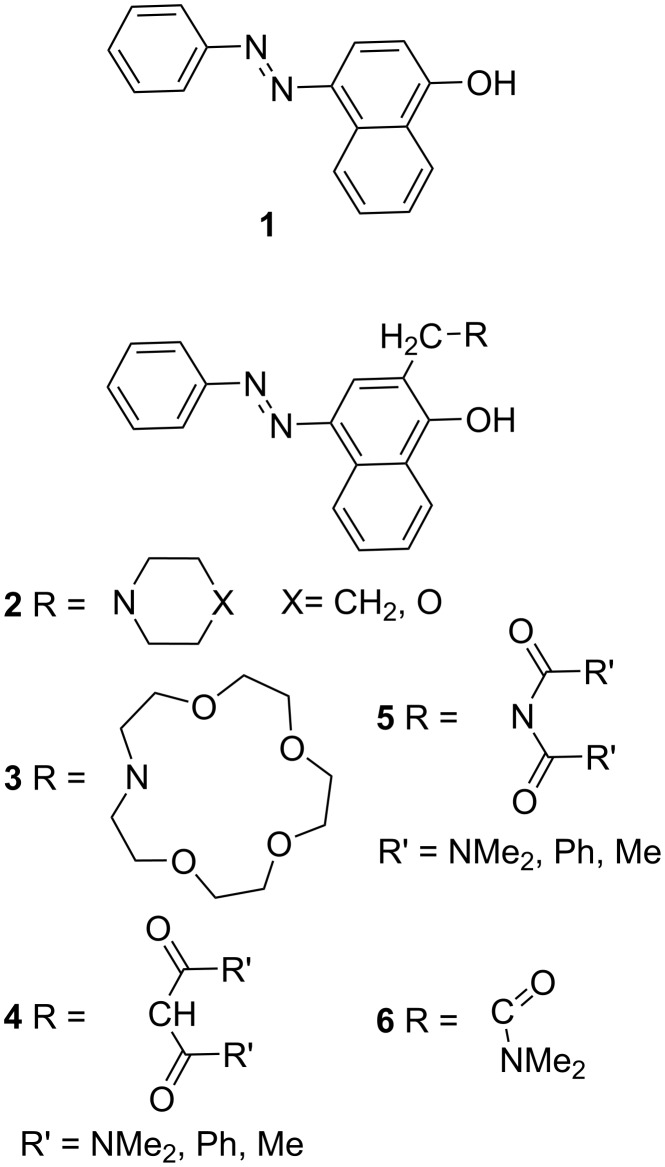
4-(Phenyldiazenyl)naphthalen-1-ol (**1**) and tautomeric ligands based on it.

Several successful tautomeric ligands, based on 4-(phenyldiazenyl)naphthalen-1-ol (**1**) [8] (**2** and **3**, Scheme 2) as a tautomeric unit have been developed by us. We found that compounds **2** and **3** exist in the neutral state solely as enol tautomers due to intramolecular hydrogen bonding involving the tautomeric hydroxy group and that the complexation shifts the equilibrium to the **K** form. Although **3** exhibits a 3D structure and as a result, shows high stability constants upon complexation, the selectivity is rather low, which can be attributed to the crown ether complexation features in general. Developing the system further, leads to modification of the ionophore part by replacing the crown ether with other ionophores, such as done in the case of **4** and **5**. The quantum-chemical calculations for **4** and **5** have demonstrated that the stable enol tautomers exist as intramolecular C=O···HO bonded system, while in the **K** forms the ionophore part does not participate in hydrogen bonding and can be considered as a basic 2-alkyl substitution [9]. Consequently, the stabilization between the **E** and **K** forms is a result of the competition between the strength of the hydrogen bonding in the enol tautomer and the effect of simple alkyl substitution in the keto form skeleton. The calculations also suggest that the efficient switching towards the enol form can be achieved only when R’ = NMe_2_ (Scheme 2).

Theoretical modelling of structures **4** and **5** have also shown that only one of the carbonyl groups from the ionophore unit really participates in the capturing of the metal ion upon complexation. Therefore, the aim of the current article is to estimate theoretically and experimentally, the tautomeric state and complexation abilities of compound **6**, where only one carbonyl group in the ionophore part is present (Scheme 2). It is expected that the enol tautomer stabilization would be achieved in the neutral state as a result of the strong intramolecular hydrogen bonding between the tautomeric OH group and the carbonyl group in the ionophore part. The complex formation, depending on the size and charge of the metal ion, should shift the tautomeric equilibrium towards the keto tautomer and should provide stabilization of the complex. To the best of our knowledge, such a system has not been synthesized and studied up to now.

## Results and Discussion

Compound **1** is a well-studied tautomeric structure featuring a moderate energy gap between the enol and the keto tautomeric forms [10]. For this reason, the tautomeric equilibrium can be easily affected by changing the solvent. However, the tautomeric equilibrium has not been switched fully to either of the tautomers in solution. For instance, the experimentally determined Δ*G* values at room temperature range from 1.42 kcal/mol, which corresponds to around 8% (in cyclohexane) or 10% (in methylcyclohexane/toluene) of the **K** tautomer [11–12], to −0.71 kcal/mol in chloroform [8], where this tautomer dominates. The Δ*G* value of 0.33 kcal/mol in acetonitrile, determined experimentally [8], have been used to validate the level of theory used in the current study. As seen from Table S1 (Supporting Information File 1) the best result has been achieved by using M06-2X/6-31++G** functional and basis set, which predicts the relative energy of the tautomers (Δ*E* value, defined as *E***_K_** − *E***_E_**) of 0.33 kcal/mol, perfectly matching the experiment.

**Figure 1:**
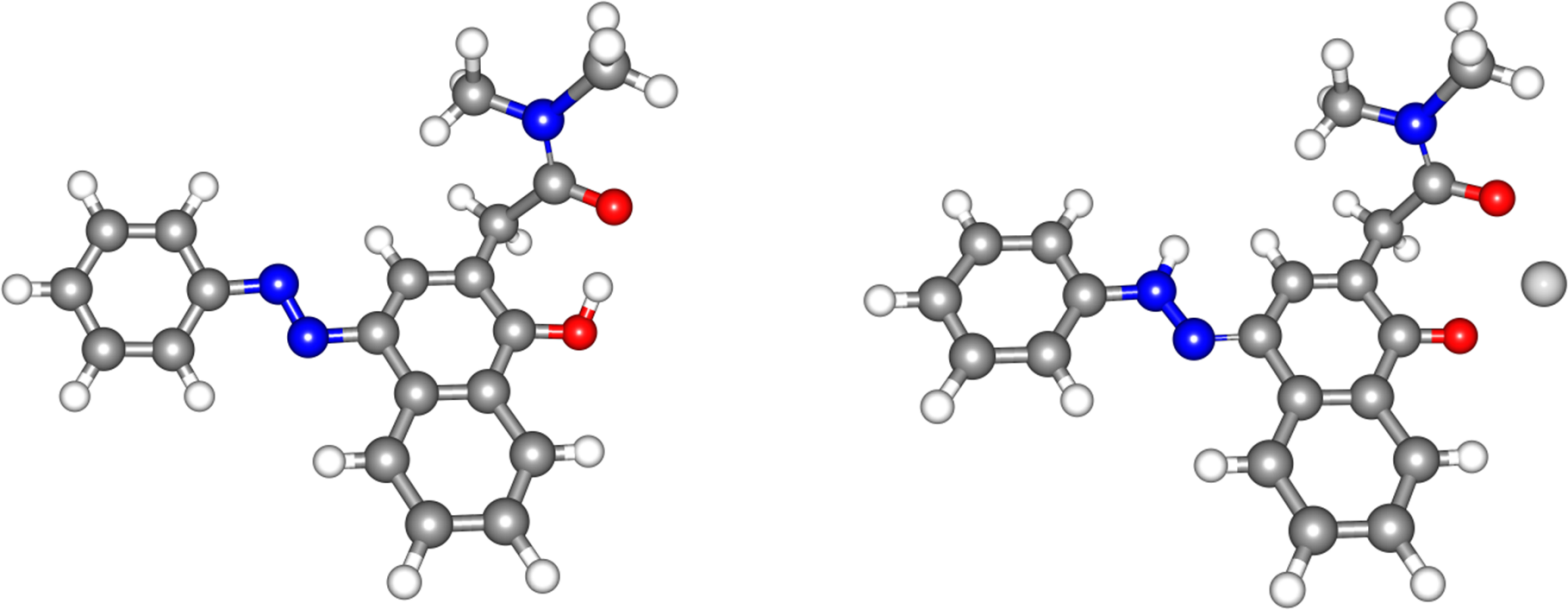
The most stable tautomeric form of **6** in neutral state (left) and upon complexation with Mg(ClO_4_)_2_ (right).

In the case of **6** the calculations yield a Δ*E* value of 3.14 kcal/mol in acetonitrile, which leads to the expectation that the tautomeric equilibrium should be fully shifted to **6E**. The corresponding most stable structure of the enol form is shown in Figure 1, where hydrogen bonding between the tautomeric OH group and the sidearm carbonyl group can be seen.

**Figure 2:**
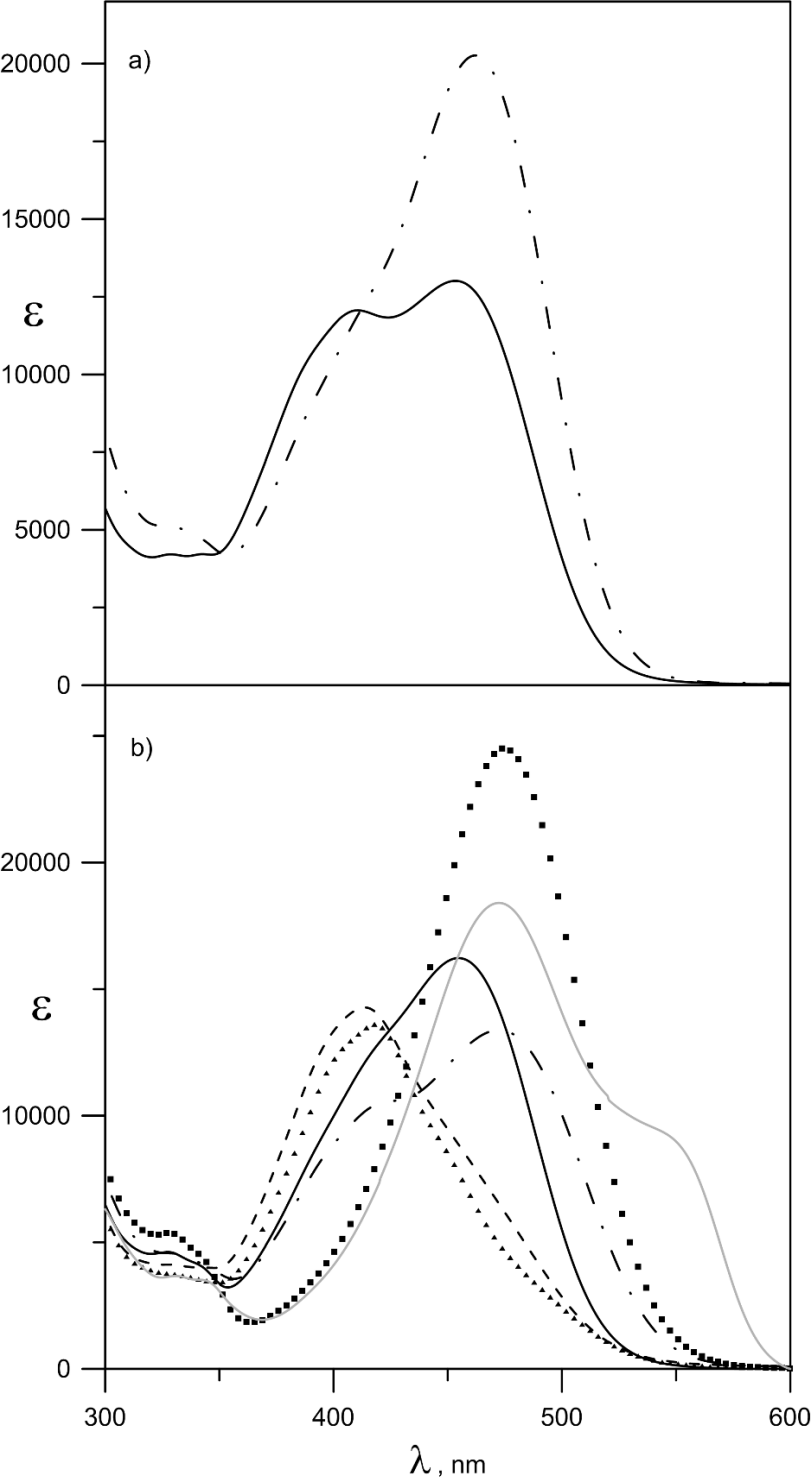
Absorption spectra of compounds **1** (a) and **6** (b) in acetonitrile (**—**), chloroform (**− · −**), dichloromethane (**---**), ethanol (■■■), toluene (▲▲▲) and dimethylformamide (grey solid line).

The tautomeric equilibrium in **1** is strongly solvent-dependent as mentioned above and which can also be seen from Figure 2a. For instance, through intermolecular hydrogen bonding with the carbonyl oxygen atom from the tautomeric backbone, chloroform stabilizes the keto tautomer, absorbing at ≈480 nm, while in acetonitrile the enol form is also presented with a maximum at ≈410 nm.

A comparison between the absorption spectra of **1** and **6** shows that the tautomeric equilibrium in **6** is also surprisingly solvent dependent. As shown on Figure 2b, the tautomeric equilibrium in **6** is shifted, but not fully, towards the **K** form in acetonitrile and chloroform and towards the **E** form in dichloromethane and toluene. In ethanol and dimethylformamide, the maximum of the enol form visually disappears (the new maximum around 550 nm in the spectrum in dimethylformamide belongs to the deprotonated form, see Figure 4 below). A careful study of the spectra shown in Figure 2b leads to the conclusion that in all solvents the absorption maximum of the enol tautomer is in the range of 415–420 nm, while the maximum of the keto form in acetonitrile (455 nm) is substantially blue shifted compared to the other solvents (≈480 nm in ethanol, dimethylformamide and chloroform).

Having in mind the theoretical predictions discussed above, the existence of the keto tautomer in solution is surprising. This behavior might mean that either the enol stabilizing intramolecular H-bonding is not strong enough and can be broken by the solvent or there are intermolecular interactions not taken into account by the calculations. The former could be the reason in some of the solvents, which have the capacity to stabilize **6K** as proton acceptor (dimethylformamide), proton donor (chloroform) or both (ethanol). The latter could be the reason for the keto tautomer stabilization in acetonitrile.

**Figure 3:**
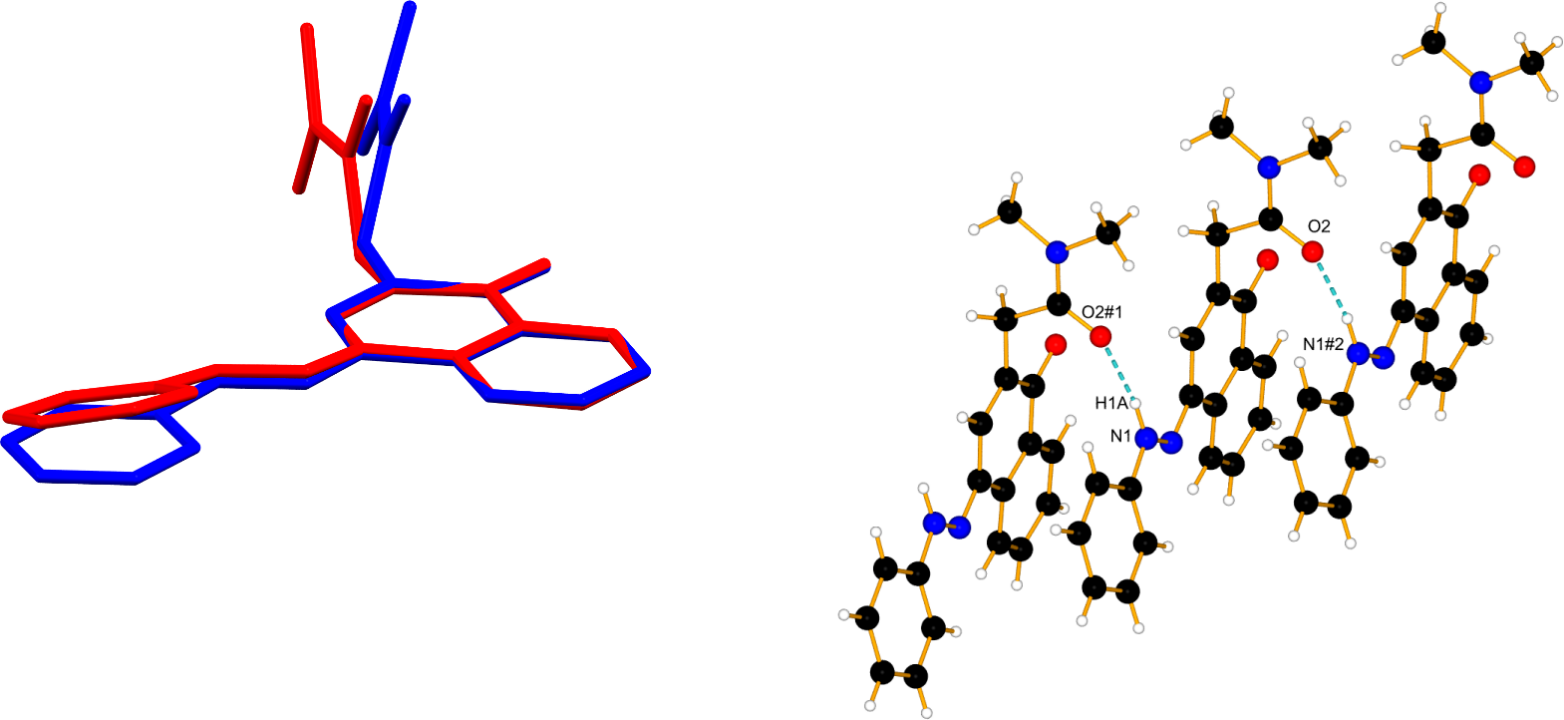
Left: Theoretically predicted structure of **6K** (blue), overlaid with the X-ray structure (red). Right: X-ray structure of **6**, #1: x − 1, y, z; #2: x + 1, y, z; H bonds are drawn as blue dashed lines.

The explanation for the sudden stabilization of **6K** was found by X-ray measurements of its crystal, obtained in acetonitrile. The crystal structure of **6**, shown in Figure 3, clearly indicates that the **K** form is stabilized through the formation of linear intermolecular associates. It can be seen that a hydrogen bond is formed between the nitrogen proton of one keto tautomer and the carbonyl group of another neighboring molecule. Probably, the process of associate formation is facilitated by the position of the chromophore part in the isolated **K** form (Figure 3, left). Obviously, the formation of the seven-membered hydrogen-bonding ring in **6E** cannot compete with the flexibility of the system in the case of the intermolecular association. Compared to another tautomeric C=O containing ionophore, recently developed [13], it seems that the existence of a carbonyl group leads in some cases to stabilization of the keto tautomer through formation of associates.

This kind of aggregation reflects to the spectrum of **6** in acetonitrile. The formed aggregate (see Figure S1, Supporting Information File 1) has a H-type structure (also called “sandwich” type) [14] with parallel assignment of the monomer molecules, consequently, its absorption maximum should be blue shifted compared to the monomeric species. If we assume that in ethanol or in dimethylformamide only the monomeric keto form is present, the blue shift of the absorption in acetonitrile indicates that the keto form here exists exclusively as H-aggregates as in the crystal structure.

**Figure 4:**
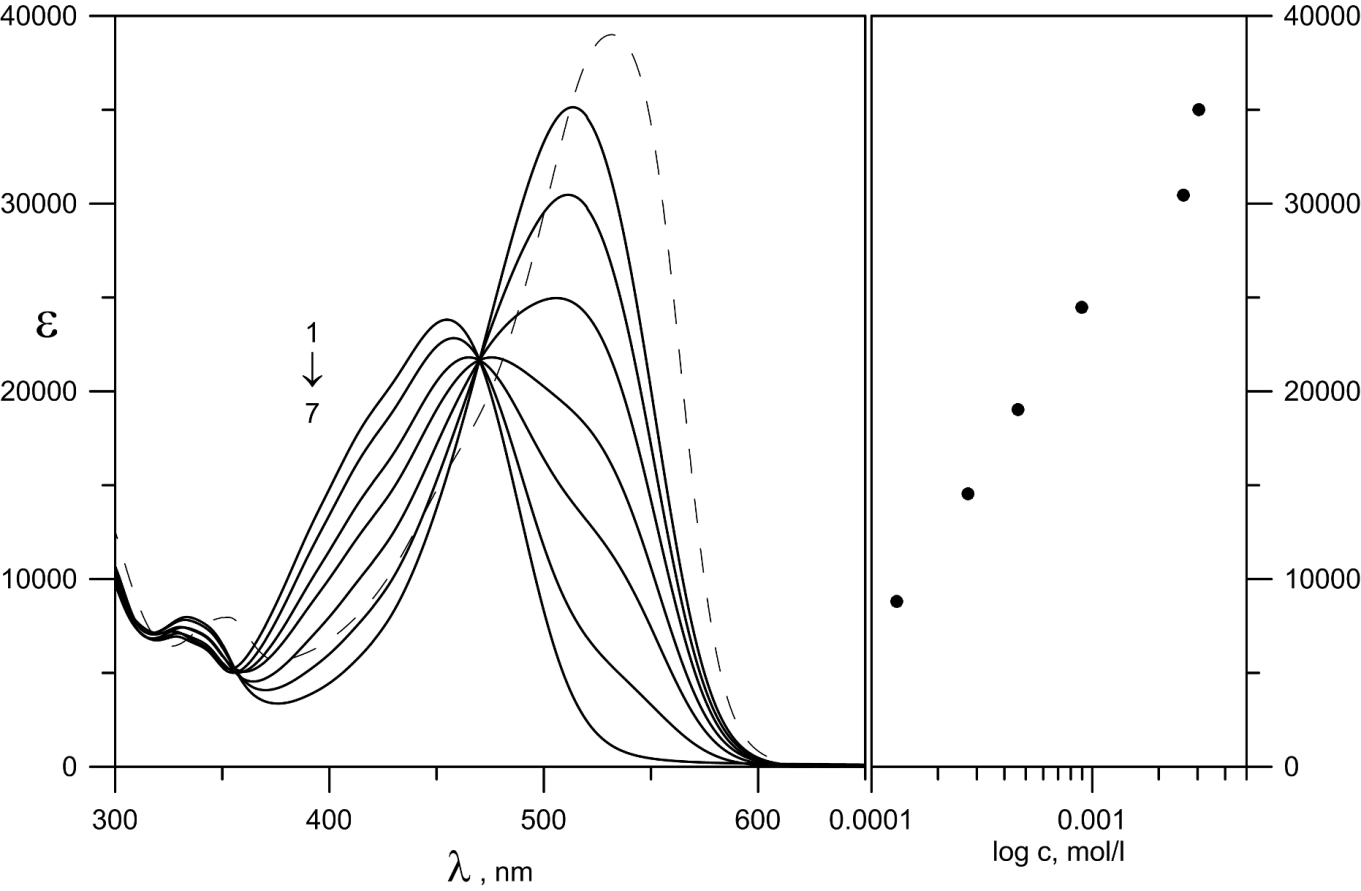
Left: Absorption spectra of **6** with stepwise addition of Mg(ClO_4_)_2_ in acetonitrile (1 – neutral ligand; 7 – complex C_Mg(ClO4)2_ = 1.23 × 10^−3^ M. The spectrum of the deprotonated form of **6** is drawn with dashes. Right: Molar absorptivity at 513 nm as a function of the concentration of the salt in a logarithmic scale.

The absorption spectra of **6** in acetonitrile upon addition of Mg(ClO_4_)_2_ are shown in Figure 4. A clear isosbestic point can be seen in the area where the enol tautomer does not absorb, indicating that the tautomeric equilibrium is shifted towards the keto tautomer (in form of the complex) as a result of the general equilibrium scheme below:


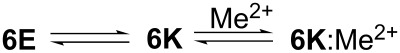


The complexation provides a substantial red shift (from 455 to 513 nm) compared to the neutral ligand with increased intensity of the new maximum at 513 nm. The comparison between the spectra of the complex and the deprotonated ligand, shown in Figure 4, indicates that the complex formation is not related to deprotonation. These results coincide with the results obtained for compound **3** [15].

**Figure 5:**
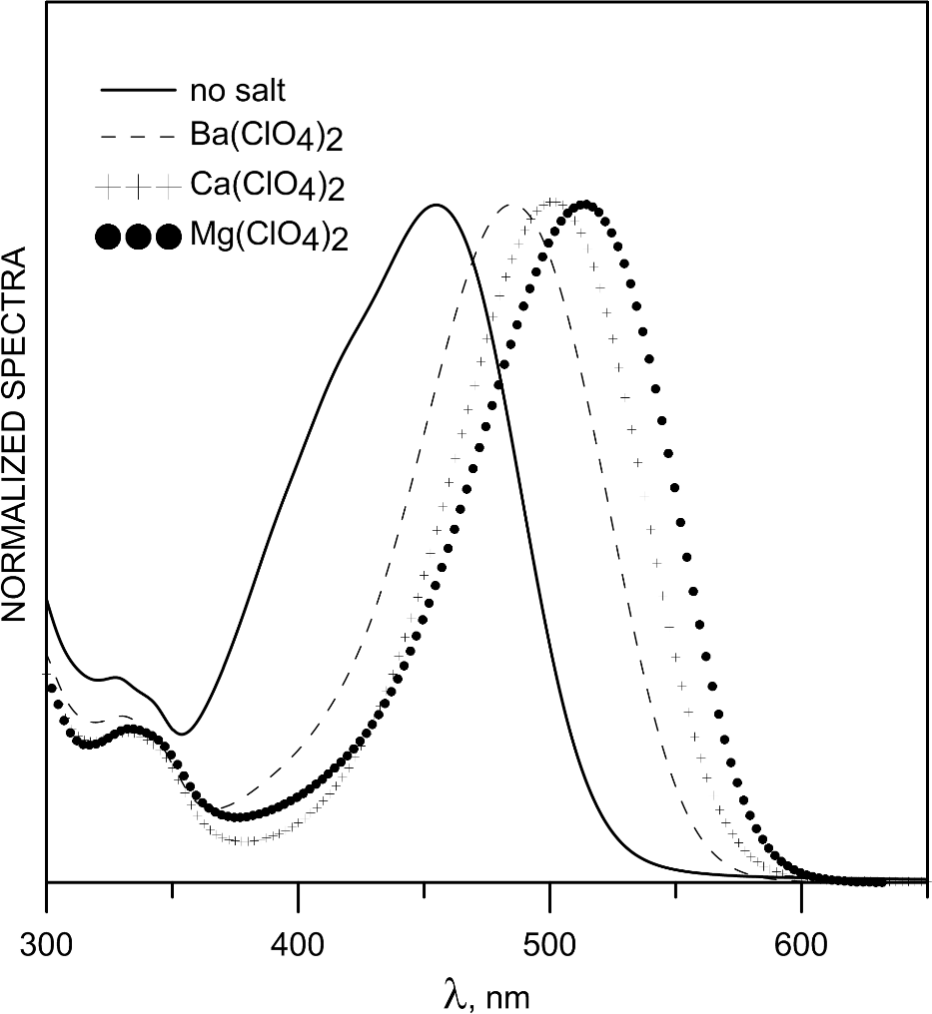
Normalized spectra of the free ligand **6** (*c* = 5 × 10^−5^ M) and its complexes obtained with Ba(ClO_4_)_2_, Ca(ClO_4_)_2_ and Mg(ClO_4_)_2_.

The complexation abilities of **6** towards some alkaline-earth metal ions were studied and the obtained spectra of the complexes are given in Figure 5. As seen the λ_max_ values of the complex changes with the change of the type of the metal ion. We assume the formation of a 1:1 complex (the Job’s plots are shown on Figure S2, Supporting Information File 1) as shown in Figure 1, which leads to a substantial red spectral shift and allows the recognition of each metal ion based on the complex peak position.

The estimated stability constants and the absorption maxima of the complexes are summarized in Table 1. The complex formation causes a substantial red shift, which varies with the metal ion. It is worth mentioning that complexation with any alkaline metal was not observed. As seen, **6** shows strong complexation with Ba^2+^, which fits well with the size of the cavity formed between the two carbonyl groups of the keto form of the ligand, while the corresponding stability constants with Ca^2+^ and Mg^2+^ are very similar. However, as shown in Figure 5 and Table 1, the difference in the spectral maxima of the complexes allows detection of each of the studied cations.

**Table 1:** Absorption maxima of the complexes with different alkaline-earth metal ions and stability constants of the complexes of **6** in acetonitrile.

Metal ion	log β	λ_max_ complex [nm]

Ba^2+^	3.2 ± 0.10	485
Ca^2+^	2.8 ± 0.04	501
Mg^2+^	2.6 ± 0.05	513

## Conclusion

In the current study, we modeled theoretically and experimentally the tautomerism and complexation abilities of a new tautomeric ligand, based on 4-(phenyldiazenyl)naphthalen-1-ol. According to the theoretical calculations the enol form stabilization could be achieved through a strong intramolecular hydrogen bond formed between the tautomeric hydroxy group and the carbonyl group from the tautomeric backbone. However, intermolecular association plays a role in some solvents as shown by the experimental results. The calculations predict that the complexation with alkali earth metal ions could lead to a full shift of the tautomeric equilibrium towards the keto tautomer, which was finally observed in solution. The formed 1:1 complexes showed large bathochromic and hyperchromic shifts in the visible spectra.

## Experimental

### Organic synthesis

**Scheme 3:**
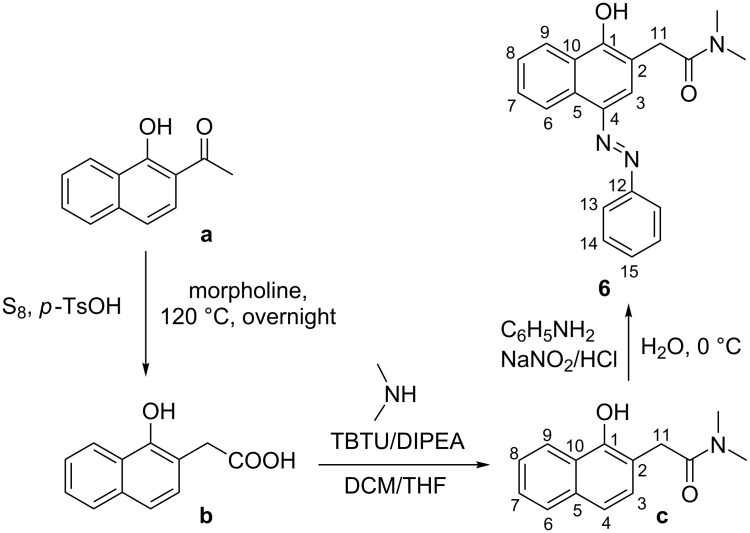
Synthetic route of **6**.

The synthetic route to compound **6** is shown in Scheme 3.

### Preparation of compound **c**

The starting intermediate **b** was prepared according to the described procedure [16] from commercially available ketone **a**.

#### 2-(1-Hydroxynaphthalen-2-yl)-*N*,*N*-dimethylacetamide (**c**)

Compound **b** (1.22 g, 6.03 mmol) was suspended in 20 mL dry dichloromethane and cooled to 0 ºC. Consequently, to this suspension were added dry diisopropylethylamine (6.3 mL, 36.20 mmol), TBTU (3.87 g, 12.07 mmol) and 2.0 M solution of dimethylamine in dry THF (6.0 mL, 12.07 mmol). The reaction mixture was stirred for 7 days at rt (reaction monitoring by TLC – dichloromethane/petroleum ether 5:2). The reaction mixture was washed successively with aq citric acid and water, dried over Na_2_SO_4_, filtered and evaporated under vacuum. The crude product was purified by column chromatography – 75 g silica gel, phase dichloromethane/petroleum ether 5:2 – to give 0.59 g (43%) of pure **c** as beige crystals. Mp 87–88 °C; ^1^H NMR (600.11 MHz, CDCl_3_, 293 K) δ 8.36 (m, 1H, H-9), 7.74 (m, 1H, H-6), 7.45 (m, 2H, H-7, H-8), 7.33 (d, *J* = 8.3 Hz, 1H, H-4), 7.13 (d, *J* = 8.3 Hz, 1H, H-3), 3.87 (s, 2H, H-11), 3.23 (s, 3H, N-C*H*_3_), 2.98 (s, 3H, N-C*H*_3_); ^13^C NMR (150.90 MHz, CDCl_3_, 293 K) δ 173.44 (1C, *C*=O), 153.26 (1C, C-1), 134.02 (2C, C-5, C-10), 128.09 (1C, C-3), 127.05 (1C, C-6), 126.18 (1C, C-7), 125.13 (1C, C-8), 122.74 (1C, C-9), 119.30 (1C, C-4), 113.11 (1C, C-2), 38.48 (1C, N-*C*H_3_), 36.89 (1C, C-11), 35.95 (1C, N-*C*H_3_); anal. calcd for C_14_H_15_NO_2_ (229.11): C, 73.34; H, 6.59; N, 6.11; found: C, 73.25; H, 6.68; N, 6.03%.

### Preparation of compound **6**

#### (*E*)-2-(1-Hydroxy-4-(phenyldiazenyl)naphthalen-2-yl)-*N*,*N*-dimethylacetamide

Preparation of phenyldiazonium salt solution: Aniline (0.90 mL, 10.00 mmol) was dissolved in a mixture of concentrated hydrochloric acid (5 mL) and distilled water (20 mL). A solution of sodium nitrite (0.83 g, 12.00 mmol) in distilled water (5 mL) was prepared in a test tube. The sodium nitrite solution was added dropwise to the acidic solution of the amine over 5 min at 0 °C. The mixture was stirred at 0 °C for 40 min. Compound **c** (0.59 g, 2.57 mmol) was dissolved in an aqueous solution of NaOH (1.03 g, 25.73 mmol in 10 mL distilled water) and cooled to 0 °C. The above prepared phenyldiazonium salt solution (6.43 mL, 2.57 mmol) was added dropwise to the solution of **c** at 0 °C. The resultant deep red mixture was stirred for 1 h at 0 °C. The crude product **6** was precipitated by addition of 20% hydrochloric acid, filtered and washed with distilled water. For further purification the crude product was dissolved in 5 mL dichloromethane and purified by column chromatography – 75 g silica gel, phase dichloromethane/methyl *tert*-butyl ether 10:1. After column chromatography, the product was additionally washed with petroleum ether and dried in vacuum to give 0.720 g (84%) of pure **6** as bright red crystals. Mp 152–153 °C; ^1^H NMR* (600.11 MHz, DMSO-*d*_6_ with 1.5 equiv excess of NaOH, 293 K) δ 11.51 (br s, 1H, O*H*), 8.50 (br d, *J* = 8.5 Hz, 1H, H-9), 8.10 (br s, 1H, H-3), 8.07 (br d, *J* = 8.0 Hz, 1H, H-6), 7.68 (m, 1H, H-8), 7.59 (m, 2H, H-13), 7.50 (m, 1H, H-7), 7.42 (m, 2H, H-14), 7.10 (m, 1H, H-15), 3.64 (s, 2H, H-11), 3.12 (s, 3H, N-C*H*_3_), 2.85 (s, 3H, N-C*H*_3_); ^13^C NMR* (150.90 MHz, DMSO-*d*_6_ with 1.5 equiv excess of NaOH, 293 K) δ 169.84 (1C, *C*=O), 135.31 (2C, C-5, C-10), 130.94 (br s, 1C, C-8), 129.41 (2C, C-14), 128.63 (br s, 1C), 126.71 (br s, 1C, C-7), 125.06 (1C, C-6), 123.67 (br s, 1C, C-15), 122.46 (1C, C-9), 115.98 (br s, 2C, C-13), 112.36 (1C, C-2), 37.16 (1C, N-*C*H_3_), 35.07 (1C, N-*C*H_3_), 34.70 (1C, C-11); anal. calcd for C_20_H_19_N_3_O_2_ (333.39): C, 72.05; H, 5.74; N, 12.60; found, C, 72.12; H, 5.70; N, 12.67%; HRMS (rel. int.) *m*/*z*: 333.14649 (−2.05527 ppm).

*Due to tautomerism, the NMR spectra in most of the solvents (DMSO-*d*_6_, CDCl_3_, acetone-*d*_6_, acetonitrile-*d*_3_ etc.) are not informative. In all cases a complicated mixture of tautomers and lack of signals was observed. Therefore, the NMR spectra were recorded in strong basic media, in order to obtain a single tautomeric skeleton. Nevertheless, some signals in the ^13^C NMR spectra do still not appear even after 1024 scans.

### Theoretical calculations

Quantum-chemical calculations were performed using the Gaussian 09 D.01 program suite [17]. The M06-2X functional [18–19] was used with the 6-31++G** basis set for the calculations. This fitted hybrid meta-GGA functional with 54% HF exchange was especially developed to describe main-group thermochemistry and noncovalent interactions. It shows very good results in predicting the position of the tautomeric equilibria for compounds with intramolecular hydrogen bonds as well as describing the ground and excited state proton transfer mechanism [20–25].

The solvent effect was described using the Polarizable Continuum Model (the integral equation formalism variant, IEFPCM, as implemented in Gaussian 09) [26]. All ground state structures were optimized without restrictions, using tight optimization criteria and an ultrafine grid in the computation of two-electron integrals and their derivatives. The true minima were verified by performing frequency calculations in the corresponding environment. The TD-DFT method [27–29], carried out with the same functional and basis set, was used for predicting vertical transitions.

### Spectral measurements

The NMR spectra were recorded on a Bruker Avance II+ 600 spectrometer. In case of CDCl_3_ tetramethylsilane was used as internal standard. In case of DMSO-*d*_6_, the spectra were calibrated to the residual solvent peaks (for DMSO-*d*_6_: δ = 2.50 for ^1^H). ^13^C NMR spectra were calibrated in all cases to the residual solvent peaks (for CDCl_3_ δ = 77.00, for DMSO-*d*_6_ δ = 39.52). The following additional NMR techniques were used for all compounds: DEPT 135, COSY, HSQC and HMBC. Mass spectra (MS) were recorded on a Thermo Scientific High Resolution Magnetic Sector MS DFS spectrometer. UV–vis spectral measurements were performed on a Jasco V-570 UV–vis–NIR spectrophotometer, equipped with a thermostatic cell holder (using Huber MPC-K6 thermostat with 1 °C precision) in spectral grade solvents at 20 °C. The complexation was studied in acetonitrile. AR grade Mg(ClO_4_)_2_ (Fluka), Ca(ClO_4_)_2_·4H_2_O (Aldrich) and Ba(ClO_4_)_2_·*x*H_2_O (Fluka) were vacuum dried at 90 °C for 3 days. Due to the red shift upon complexation, the estimation of the stability constants was performed at the maximum of the complex using the final complex spectrum (Figure 5). Deprotonation was made with trimethylamine (Aldrich).

### X-ray crystallographic measurements

#### Experimental

Single crystals of **6** were crystallized from acetonitrile by slow evaporation. A suitable crystal was selected and was mounted on a loop in oil on a Stoe IPDS2T diffractometer. The crystallographic data of the single crystal were collected with Cu Kα_1_ radiation (λ = 1.54186 Å). The crystal was kept at 250(2) K during data collection by an Oxford Cryosystem open-flow cryostat. Using Olex2 [30], the structure was solved with the ShelXT [31] structure solution program using Intrinsic Phasing and refined with the ShelXL [31] refinement package using Least Squares minimization.

#### Crystal structure determination of **6**

Crystal data for C_20_H_19_N_3_O_2_ (*M* =333.38 g/mol): monoclinic, space group *P*2_1_/*c* (no. 14), *a* = 5.5438(8) Å, *b* = 17.850(2) Å, *c* = 17.184(3) Å, β = 91.719(12)°, *V* = 1699.7(4) Å^3^, *Z* = 4, *T* = 250(2) K, μ(Cu Kα) = 0.691 mm^−1^, *d*_calc_ = 1.303 g/cm^3^, 14285 reflections measured (7.142° ≤ 2Θ ≤ 135.89°), 2929 unique (*R*_int_ = 0.0894, *R*_sigma_ = 0.0584) which were used in all calculations. The final *R*_1_ was 0.0627 (I > 2σ(I)) and *wR*_2_ was 0.1642 (all data). The CIF file can be obtained from the Cambridge Crystallographic Data Centre: CCDC-1914884 (**6**).

## Supporting Information

File 1 Additional experimental and calculated data.
