# Supplementary material for: Tautomerism as primary signaling mechanism in metal sensing: the case of amide group
Source: Beilstein J Org Chem. 2019 Aug 8;15:1898–906. doi: 10.3762/bjoc.15.185 (PMC6693416; doi:10.3762/bjoc.15.185)
Supplement: File 1 — Additional experimental and calculated data. [file Beilstein_J_Org_Chem-15-1898-s001.pdf]

BEILSTEIN JOURNAL OF ORGANIC CHEMISTRY

Supporting Information

for

Tautomerism as primary signaling mechanism in metal sensing: the case of amide group

Vera Deneva, Georgi Dobrikov, Aurelien Crochet, Daniela Nedeltcheva, Katharina M. Fromm and Liudmil Antonov

Beilstein J. Org. Chem. **2019,** *15,* 1898–1906. doi:10.3762/bjoc.15.185

Additional experimental and calculated data

License and Terms: This is a supporting information file under the terms of the Creative Commons Attribution License (http://creativecommons.org/licenses/by/4.0). Please note that the reuse, redistribution and reproduction in particular requires that the authors and source are credited.
The license is subject to the *Beilstein Journal of Organic Chemistry* terms and conditions: (https://www.beilstein-journals.org/bjoc)

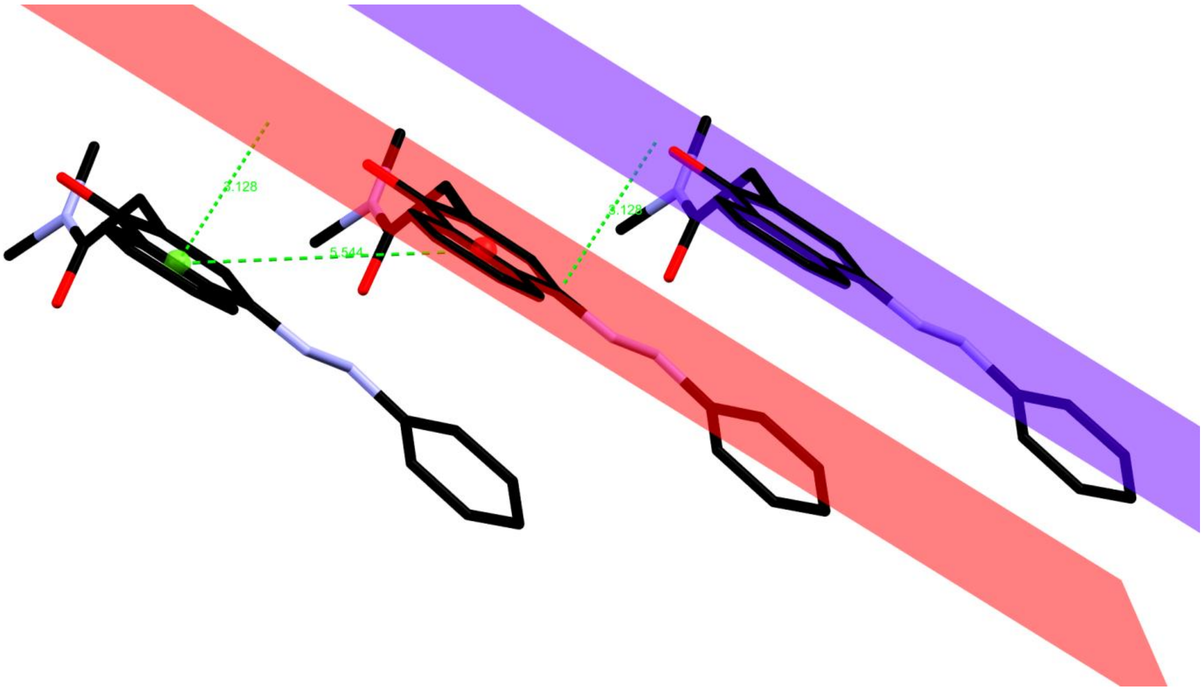

Figure S1: Distance between monomers in the aggregate crystal structure.

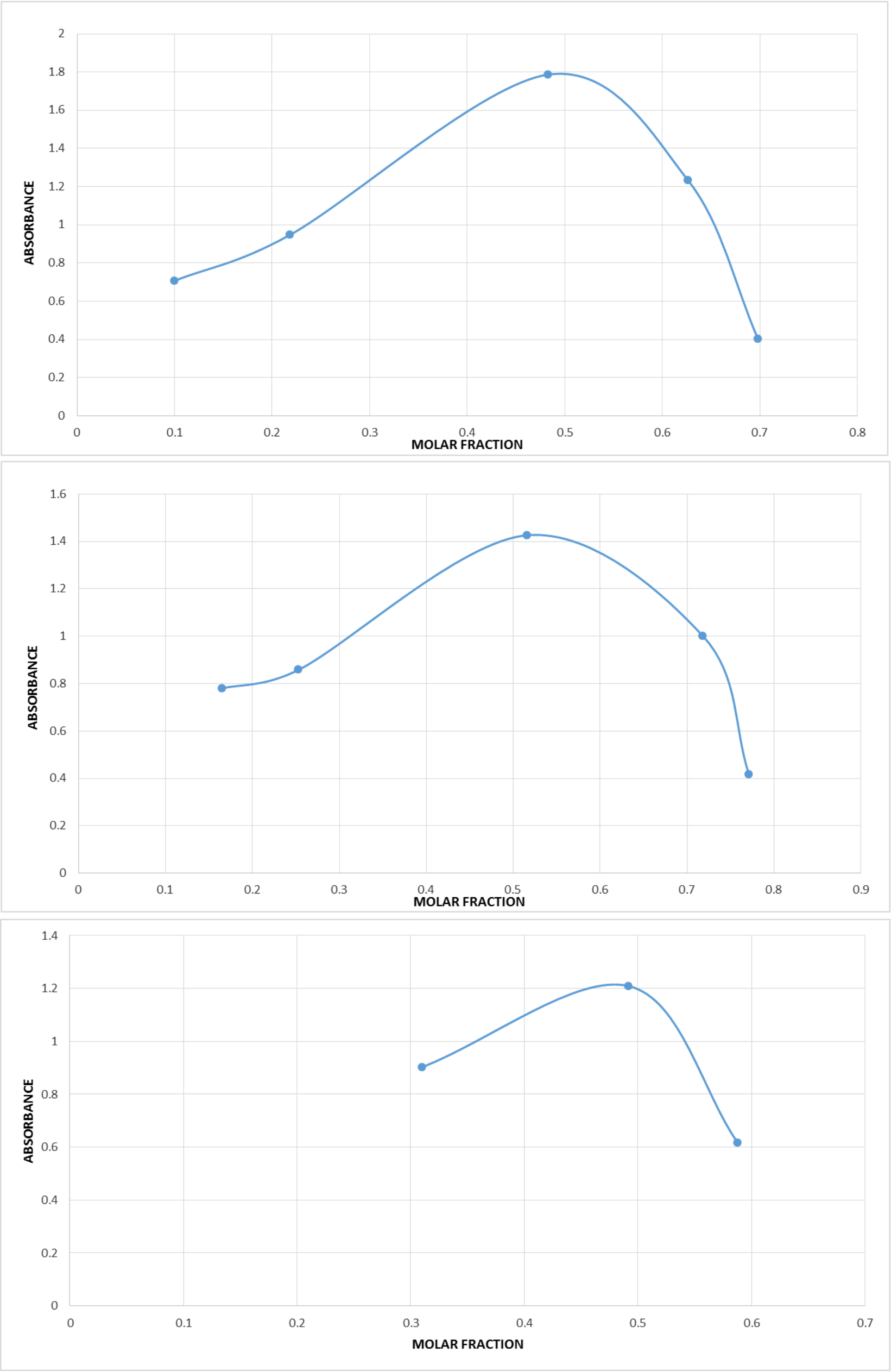

Figure S2: Job's plots of the complexes of **6** with Mg^{2+}, Ca^{2+} and Ba^{2+}. Molar fractions are calculated as: $X_L = C_L/(C_M + C_L)$, where C_L is the concentration of the ligand and C_M is the concentration of the metal ion.

Table S1: Relative stability[a] of the tautomers, M062X in kcal/mol for **1** in acetonitrile[b].

Level of theory	ΔE, [kcal/mol]
6-31++G**	0.33
6-311++G**	-0.24
cc-pVTZ	-0.36
TZVP	-0.40
def2TZVP	-0.85

[a]Positive value, means more stable enol form and vice versa.

[b]The experimentally determined value is 0.33 kcal/mol [1].

Reference

1. Fabian, W. M. F., Antonov, L., Nedeltcheva, D., Kamounah, F. S.; Taylor, P. J. *J. Phys. Chem. A* **2004,** *108,* 7603–7612.
